# Comparison of Clinical Efficacy and Safety Between da Vinci Robotic and Laparoscopic Intersphincteric Resection for Low Rectal Cancer: A Meta-Analysis

**DOI:** 10.3389/fsurg.2021.752009

**Published:** 2021-12-02

**Authors:** Jie Zhang, Xingshun Qi, Fangfang Yi, Rongrong Cao, Guangrong Gao, Cheng Zhang

**Affiliations:** ^1^Department of General Surgery, General Hospital of Northern Theater Command (Formerly Called General Hospital of Shenyang Military Area), Shenyang, China; ^2^Postgraduate College, Dalian Medical University, Dalian, China; ^3^Department of Gastroenterology, General Hospital of Northern Theater Command (Formerly Called General Hospital of Shenyang Military Area), Shenyang, China; ^4^Postgraduate College, Jinzhou Medical University, Jinzhou, China

**Keywords:** da Vinci robot, intersphincteric resection, laparoscope, low rectal cancer, clinical efficacy

## Abstract

**Background and Aims:** The intersphincteric resection (ISR) is beneficial for saving patients' anus to a large extent and restoring original bowel continuity. Laparoscopic ISR (L-ISR) has its drawbacks, such as two-dimensional images, low motion flexibility, and unstable lens. Recently, da Vinci robotic ISR (R-ISR) is increasingly used worldwide. The purpose of this article is to compare the feasibility, safety, oncological outcomes, and clinical efficacy of R-ISR vs. L-ISR for low rectal cancer.

**Methods:** PubMed, EMBASE, Cochrane Library, and Web of Science were searched to identify comparative studies of R-ISR vs. L-ISR. Demographic, clinical, and outcome data were extracted. Mean difference (MD) and risk ratio (RR) with their corresponding confidence intervals (CIs) were calculated.

**Results:** Five studies were included. In total, 510 patients were included, of whom 273 underwent R-ISR and 237 L-ISR. Compared with L-ISR, R-ISR has significantly lower estimated intraoperative blood loss (MD = −23.31, 95% CI [−41.98, −4.64], *P* = 0.01), longer operative time (MD = 51.77, 95% CI [25.68, 77.86], *P* = 0.0001), hospitalization days (MD = −1.52, 95% CI [−2.10, 0.94], *P* < 0.00001), and postoperative urinary complications (RR = 0.36, 95% CI [0.16, 0.82], *P* = 0.02).

**Conclusions:** The potential benefits of R-ISR are considered as a safe and feasible alternative choice for the treatment of low rectal tumors.

## Introduction

According to recent cancer statistics, colorectal cancer is the third most common malignancy (1). However, 75% of rectal cancer is low rectal cancer, which is usually defined as the lower rectum within 5 cm from the anal verge (2). Surgery is considered the first choice for low rectal cancer. The treatment goal for surgeons is to preserve anal function under the premise of tumor resection in low rectal cancer. Abdominoperineal resection has been the standard surgery for advanced low rectal cancer for over a century, but its efficacy was less than satisfactory, resulting in a permanent colostomy, which greatly influences the patient's quality of life (3). In recent years, several new techniques have emerged aiming to preserve anal function under the premise of tumor resection in low rectal cancer. Intersphincteric resection (ISR) is one of the new operations, based on the dissection of the anatomical plane between the internal anal sphincter and the external anal sphincter, making it possible to increase the preservation of the sphincter and avoid a permanent colostomy (4, 5).

The laparoscope has the effect of magnifying the field of vision, which is more clear than open surgery. It can avoid the blindness of resection of the low pelvic tumor. In addition, it can also avoid tumor implantation caused by compression (6–8). Meanwhile, in many studies, laparoscope had lower blood loss, less analgesics, better recovery speed and quality, earlier restoration of intestinal function, and shorter hospital stay as compared with open surgery (6–8). However, laparoscope has its drawbacks, such as two-dimensional images, low motion flexibility, and unstable lens. For obese patients and male patients with pelvic stenosis, laparoscopic visual field exposure and operation space are particularly limited, which not only makes the anatomy difficult, but also easily damages the pelvic autonomic nerve during operation. In addition, the surgeons have to stand for a long time during the operation, which increases their fatigue. At the same time, laparoscopic surgery requires the coordination of the operator and the lens holder. These objective factors have limited the development of laparoscopic ISR (L-ISR). By comparison, da Vinci robotic ISR (R-ISR) has more advantages, such as three-dimensional vision, tremor filtering, flexible EndoWrist instruments, and better ergonomics to reduce fatigue (9–12).

The purpose of this article is to compare the feasibility, safety, clinical efficacy, and short-term oncological outcomes of L-ISR vs. R-ISR for the treatment of low rectal cancer.

## Methods

### Registration

This meta-analysis was registered on the PROSPERO database and performed in accordance with the preferred reporting items for systematic review and meta-analysis (PRISMA) guidelines (13). The registration number of PROSPERO was CRD42021265545.

### Search Strategy

The relevant publications were searched *via* PubMed, EMBASE, Cochrane library, and Web of Science databases. The search items were as follows: (rectal neoplasms OR rectal cancer OR rectal adenocarcinoma OR rectal tumor OR rectum cancer OR rectum adenocarcinoma OR rectum tumor) AND (da Vinci robot OR da Vinci OR robotics OR robot OR robotic OR robotically OR robot-assisted OR robotic-assisted) AND (laparoscopy OR laparoscope OR laparoscopic) AND (ISR OR internal sphincterectomy OR intersphincteric resection). The date of the last search was July 20, 2021.

### Study Selection

The inclusion criteria were as follows: (1) Patients should be histologically diagnosed with low rectal cancer; (2) R-ISR should be the treatment choice in the experimental group, and L-ISR should be the treatment choice in the control group; (3) studies should provide the data regarding feasibility, safety, clinical efficacy, and/or short-term oncological outcomes; and (4) the publication language was not limited. The exclusion criteria were as follows:(1) duplicate articles; (2) review articles; (3) comments and correspondences; (4) meta-analyses; (5) irrelevant topics; (6) case reports; (7) unable to extract the data regarding patients with low rectal cancer; and (8) overlapping data.

### Data Extraction

Data were extracted from the included studies by two reviewers independently. The following data were extracted, including first author, publication year, regions, number of patients, age, gender, BMI, American Society of Anesthesiologists (ASA) score, proportion of radiotherapy and chemotherapy, distance from the tumor to the anus, intraoperative blood loss, operative time, lymph node harvest, circumferential resection margin, distal resection margin, conversion rate, time to first flatus, time to postoperative diet, duration of hospital stay, postoperative complications, anastomotic leakage, postoperative ileus, postoperative urinary complications, and intra-abdominal abscess.

### Study Quality Assessment

The Newcastle-Ottawa Scale (NOS) was used to evaluate the quality of non-randomized studies. The scale consists of three parts, namely, selection of research subjects (4 points), intergroup comparability (2 points), and outcome measurement (3 points). The highest score should be 9 points. A score of <6 points is considered to be of low quality, while a score of ≥6 points is considered to be of high quality.

### Statistical Analysis

The difference was compared between L-ISR vs. R-ISR for the treatment of low rectal cancer. Only a random-effect model was employed. Continuous data were expressed as mean difference (MD) with a 95% CI as the effect size. For dichotomous variables, pooled risk ratios (RRs) with 95% CI were calculated to assess the treatment efficacy. *P* < 0.05 was considered as a statistically significant difference. The heterogeneity was evaluated by the *I*^2^ statistics and chi-square test. *I*^2^ > 50% and/or *P* < 0.1 were considered to have a statistically significant heterogeneity. Publication bias was not assessed by the funnel plot due to a small number of included studies. Data were analyzed using the Review Manager Version 5.4 (Cochrane collaboration, the Nordic Cochrane Centre, Copenhagen, Denmark).

## Results

### Study Selection

**Figure 1:**
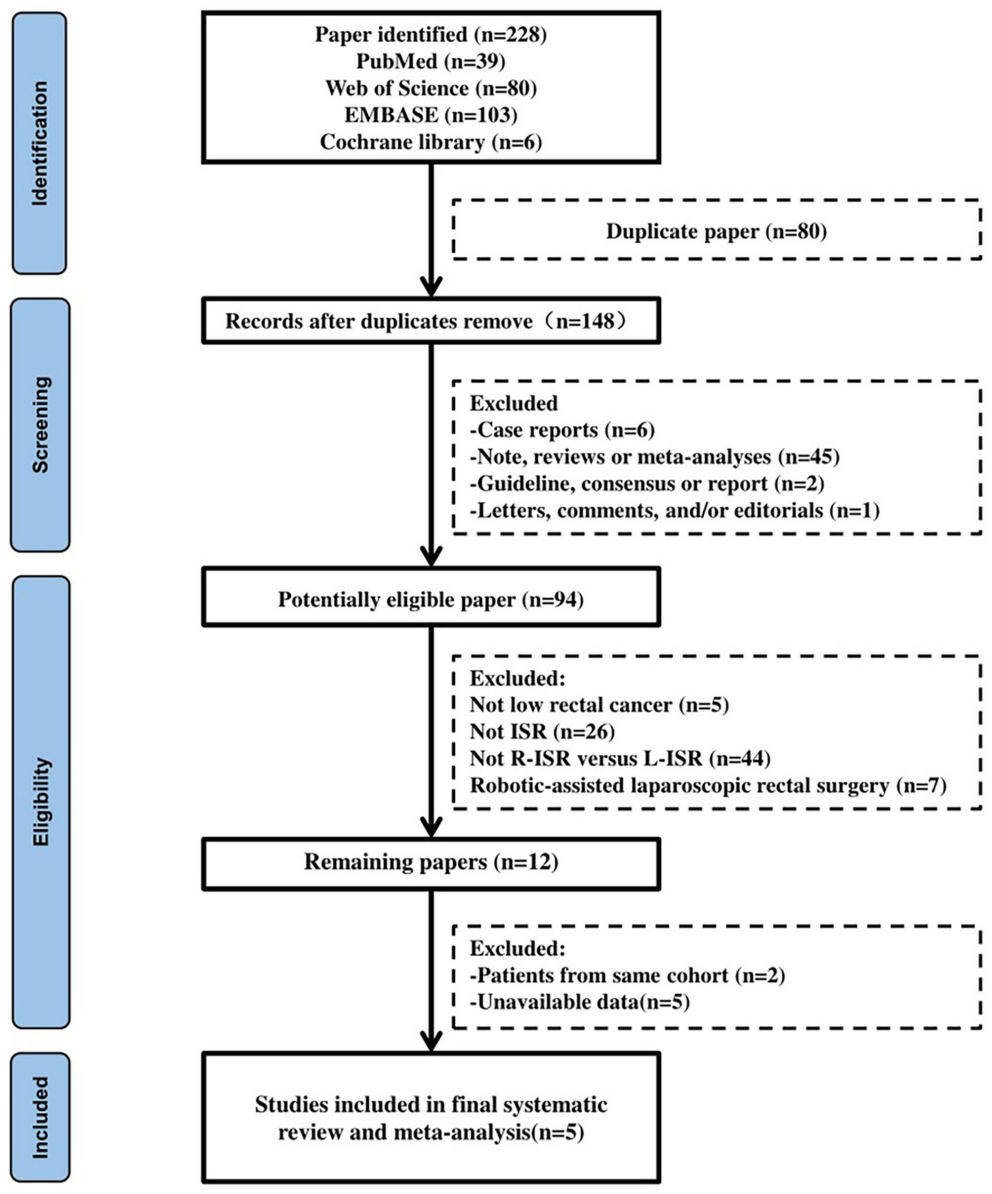
Flow diagram of study inclusion.

A total of 228 articles were identified: 39 articles in the PubMed database, 103 articles in EMBASE database, 6 articles in the Cochrane Library database, and 80 papers in Web of Science. Five studies were finally included (Figure 1) (14–18). All five studies were of retrospective nature. Four studies were conducted in Korea, and one study in Taiwan. The characteristics of studies are shown in Table 1.

**Table 1:** Characteristics of included studies.

**References**	**Setting**		**Study Design**	**Enrollment period**		**Patients, n**	
	**Country**	**Institution**		**Start**	**End**	**R-ISR**	**L-ISR**
Baek et al. (14)	Korea	Single	Retrospective cohort study	2007.01	2010.12	47	**37**
Park et al. (16)	Korea	Single	Retrospective cohort study	2008.03	2011.03	40	40
Kuo et al. (18)	Korea	Single	Retrospective cohort study	2009.11	2013.07	36	28
Park et al. (17)	Korea	Multi	Retrospective cohort study	2008.01	2011.05	106	106
Yoo et al. (15)	Taiwan, China	Single	Retrospective cohort study	2006.09	2011.08	44	26

*R-ISR, robotic intersphincteric resection; L-ISR, laparoscopic intersphincteric resection*.

### Characteristics of Study Participants

A total of 510 patients were analyzed: 273 patients underwent R-ISR and 237 patients L-ISR. The sample size varied from 26 to 106 among these studies, and 68.6% (350/510) of patients were men. With respect to the chemoradiotherapy, 63% (172/273) of patients undergoing R-ISR and 42.6% (101/207) of patients undergoing L-ISR were treated by chemoradiotherapy, respectively. The characteristics of patients are shown in Table 2.

**Table 2:** Patient characteristics.

**References**	**Age**	**Male**	**BMI**	**Chemoradiotherapy**	**Tumor stage, T0–T2, %**		**Tumor stage, T3–T4, %**		**ASA score**		**Distanced from the anal margin**
	**(R-ISR/L-ISR, years)**	**(R-ISR**	**(R-ISR/L-ISR, kg/m2)**	**(R-ISR**	**R-ISR**	**L-ISR**	**R-ISR**	**L-ISR**	**R-ISR (I/II/III)**	**L-ISR (I/II/III)**	**(R-ISR/L-ISR, cm)**
		**/L-ISR)**		**/L-ISR)**							
Baek et al. (14)	58.0 ± 12.9	31/28	23.37 ± 3.27	20/12	76.6	70.2	23.4	29.7	22/24/1	25/12	4.39 ± 2.25
	/61.8 ± 12.8		/23.4 ± 2.73								/5.52 ± 3.74
Park et al. (16)	57.3 ± 12.1	28/25	23.9 ± 2.4	32/20	50.0	35.0	50.0	65.0	27/9/4	24/14/2	3.4 ± 1.1
	/63.6 ± 10.6		/24.3 ± 3.1								/3.6 ± 1.3
Kuo et al. (18)	55.9 (30–89)	21/17	23.78/23.32 (median)	28/28	16.7	10.7	83.3	89.3	0/33/3	4/22/2	3.83 (1.5–5.0)
	/54.9 (25–88)										/3.71 (2.0–6.0)
Park et al. (17)	59.6 ± 10.8	75/71	24.3 ± 2.8	68/60	55.7	54.7	44.3	45.3	48/52/6	42/50/14	3.2 ± 1.0
	/61.7 ± 9.6		/23.8 ± 3.3								/3.3 ± 1.1
Yoo et al. (15)	59.77 ± 12.33	35/19	24.13 ± 3.33	24/7	38.6	26.9	61.4	73.1	26/17/1	15/11	3.24 ± 0.78
	/60.5 ± 10.75		/21.42 ± 3.13								/3.71 ± 0.89

### Study Quality

The study quality assessment is shown in Table 3. All of the five studies were of high quality.

**Table 3:** Newcastle-Ottawa Scale for bias risk assessment of non-randomized studies.

**Study**	**Selection**				**Comparability**	**Outcomes**			**Total**
	**Representativeness of the exposed cohort**	**Selection of the non-exposed cohort**	**Ascertainment of exposure**	**Definition that outcome of interest was not present at the start of study**		**Ascertainment of outcome**	**Was follow-up long enough for outcomes to occur**	**Adequacy of follow-up of cohorts**	
Park et al. (16)	1	1	1	1	2	1	0	0	7
Baek et al. (14)	1	1	1	1	1	1	0	0	6
Kuo et al. (18)	1	1	1	1	1	1	1	1	8
Yoo et al. (15)	1	1	1	1	1	1	0	1	7
Park et al. (17)	1	1	1	1	1	1	0	1	7

### Meta-Analyses

#### Intraoperative Blood Loss

**Figure 2:**
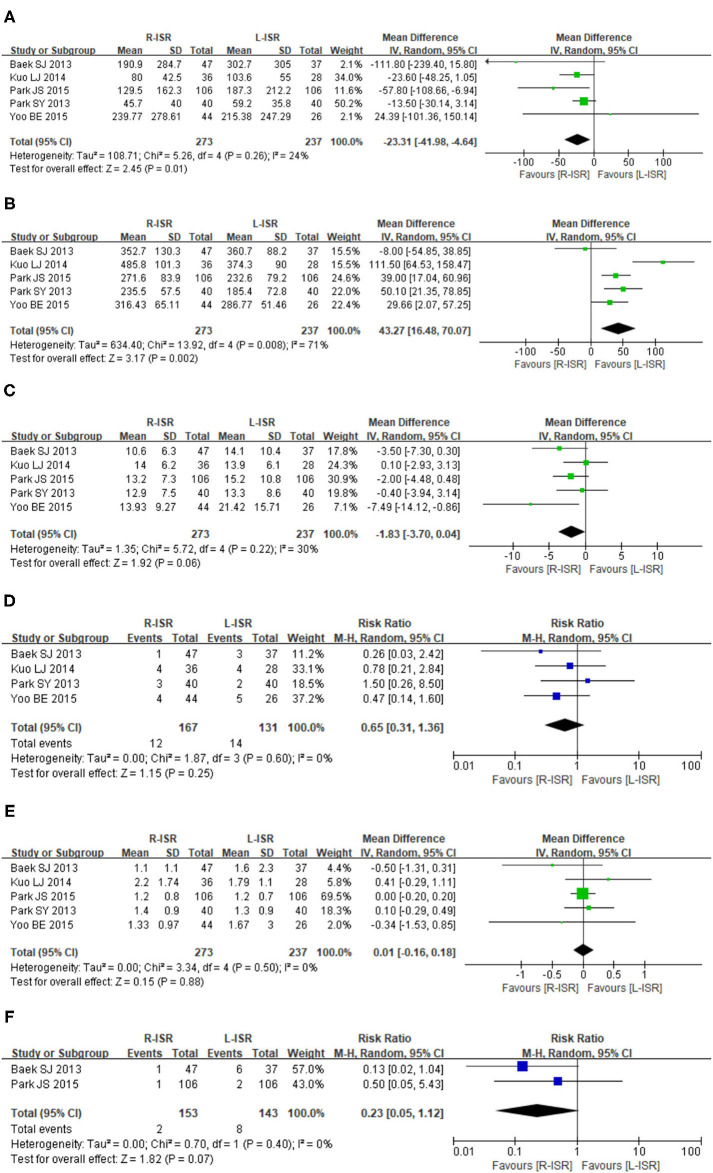
Forest plots of perioperative outcomes comparing intraoperative blood loss **(A)**, operative time **(B)**, the number of retrieved lymph nodes **(C)**, circumferential resection margin **(D)**, distal resection margin **(E)**, and conversion rate **(F)**.

Intraoperative blood loss was significantly lower in patients undergoing R-ISR than in those undergoing L-ISR (MD = −23.31, 95% CI [−41.98, −4.64], *P* = 0.01) (Figure 2A). Among the studies, the heterogeneity was not significant (*I*^2^ = 24%, *P* = 0.26).

#### Operative Time

Operative time of R-ISR was significantly longer than that of L-ISR (MD = 51.77, 95% CI [25.68, 77.86], *P* = 0.0001) (Figure 2B). Among the studies, the heterogeneity was significant (*I*^2^ = 68%, *P* = 0.03).

#### Number of Retrieved Lymph Nodes

The number of lymph node harvested was not significantly different between patients undergoing R-ISR and L-ISR (MD = −1.83, 95% CI [−3.70, 0.04], *P* = 0.06) (Figure 2C). Among the studies, the heterogeneity was not significant (*I*^2^ = 30%, *P* = 0.22).

#### Circumferential Resection Margin

Circumferential resection margin was not significantly different between patients undergoing R-ISR and L-ISR (RR = 0.65, 95% CI [0.31, 1.36], *P* = 0.25) (Figure 2D). Among the studies, the heterogeneity was not significant (*I*^2^ = 0%, *P* = 0.60).

#### Distal Resection Margin

Distal resection margin was not significantly different between patients undergoing R-ISR and L-ISR (MD = 0.01, 95% CI [−0.16, 0.18], *P* = 0.88) (Figure 2E). Among the studies, the heterogeneity was not significant (*I*^2^ = 0%, *P* = 0.50).

#### Conversion Rate

Conversion rate was not significantly different between patients undergoing R-ISR and L-ISR (RR = 0.23, 95% CI [0.05, 1.12], *P* = 0.07) (Figure 2F). Among the studies, the heterogeneity was not significant (*I*^2^ = 0%, *P* = 0.40).

#### Time to First Flatus

**Figure 3:**
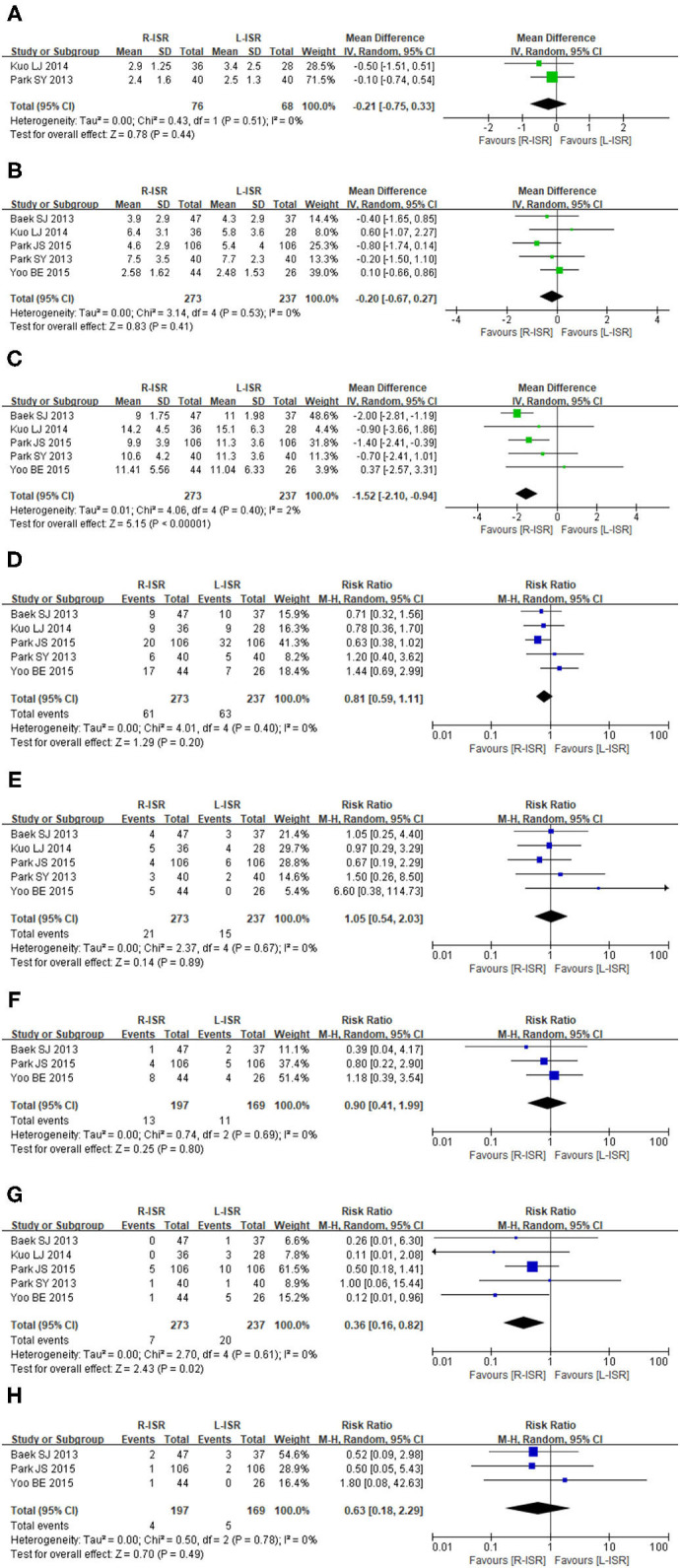
Forest plots postoperative outcomes comparing time to first flatus **(A)**, time to resume regular diet **(B)**, duration of hospital stay **(C)**, postoperative complications **(D)**, anastomotic leakage **(E)**, postoperative ileus **(F)**, postoperative urinary complications **(G)**, intra-abdominal abscess **(H)**.

Time to first flatus was not significantly different between patients undergoing R-ISR and L-ISR (MD = −0.21, 95% CI [−0.75, 0.33], *P* = 0.44) (Figure 3A). Among the studies, the heterogeneity was not significant (*I*^2^ = 0%, *P* = 0.51).

#### Time to Resume a Regular Diet

Time to resume regular diet was not significantly different between patients undergoing R-ISR and L-ISR (MD = −0.20, 95% CI [−0.67, 0.27], *P* = 0.41) (Figure 3B). Among the studies, the heterogeneity was not significant (*I*^2^ = 0%, *P* = 0.53).

#### Duration of Hospital Stay

Duration of hospital stay was significantly lower in patients undergoing R-ISR than in those undergoing L-ISR (MD = −1.52, 95% CI [−2.10, 0.94], *P* < 0.00001) (Figure 3C). Among the studies, the heterogeneity was not significant (*I*^2^ = 2%, *P* = 0.40).

#### Postoperative Complications

The incidence of postoperative complications was not significantly different between patients undergoing R-ISR and L-ISR (RR = 0.81, 95% CI [0.59, 1.11], *P* = 0.2) (Figure 3D). Among the studies, the heterogeneity was not significant (*I*^2^ = 1%, *P* = 0.40).

#### Anastomotic Leakage

The incidence of anastomotic leakage was not significantly different between patients undergoing R-ISR and L-ISR (RR = 1.05, 95% CI [0.54, 2.03], *P* = 0.89) (Figure 3E). Among the studies, the heterogeneity was not significant (*I*^2^ = 0%, *P* = 0.67).

#### Postoperative Ileus

The incidence of postoperative ileus was not significantly different between patients undergoing R-ISR and L-ISR (RR = 0.90, 95% CI [0.41, 1.99], *P* = 0.80) (Figure 3F). Among the studies, the heterogeneity was not significant (*I*^2^ = 0%, *P* = 0.69).

#### Postoperative Urinary Complications

The incidence of postoperative urinary complications was significantly lower in patients undergoing R-ISR than in those undergoing L-ISR (RR = 0.36, 95% CI [0.16, 0.82], *P* = 0.02) (Figure 3G). Among the studies, the heterogeneity was not significant (*I*^2^ = 0%, *P* = 0.61).

#### Intra-Abdominal Abscess

The incidence of intra-abdominal abscess was not significantly different between patients undergoing R-ISR and L-ISR (RR = 0.63, 95% CI [0.18, 2.29], *P* = 0.49) (Figure 3H). Among the studies, the heterogeneity was not significant (*I*^2^ = 0%, *P* = 0.78).

## Discussion

This meta-analysis has several following findings: (1) R-ISR had significantly lower estimated intraoperative blood loss and risk of postoperative urinary complications, shorter duration of hospitalization, and longer operative time than L-ISR. (2) There was no significant difference in number of retrieved lymph nodes, circumferential resection margin, distal resection margin, conversion rate, time to first flatus, time to resume regular diet, postoperative complications, anastomotic leakage, postoperative ileus, or intra-abdominal abscess between the two groups.

Previous studies have suggested that the amount of blood loss is an independent risk factor for postoperative adverse events, cancer recurrence, and poorer overall survival (19, 20). In our meta-analysis, intraoperative blood loss was significantly lower in the R-ISR group than in the L-ISR group. This is because da Vinci robot has more advantages, such as three-dimensional vision, tremor filtering, and a 7-degree of EndoWrist instrument. Such benefits provide an access to the narrow pelvis with articulating instruments and identify blood vessels and clear lymph nodes in the surgical area more clearly as compared with laparoscope (21). Furthermore, by reducing blood loss, R-ISR is helpful for improving postoperative recovery and may allow greater preservation of immune function in cancer patients, possibly thereby enhancing anti-neoplasm immune response and reducing the risk of tumor progression (22).

Our meta-analysis showed that the operative time was significantly longer in patients undergoing R-ISR than in those undergoing L-ISR. This is mainly because robotic surgery requires the docking robot and the replacement of the robotic EndoWrist (23). However, the recently invented Xi system's multi-quadrant capability can shorten the operation time by reducing redocking. In addition, the operative time is related to the skills of the surgeons. Kuo et al. showed that the mean time to complete robotic surgery was 519.5 min in the first 19 cases and only 448.2 min in the last 17 cases (24). Therefore, the operative time can be gradually decreased with increased surgeons' experiences, especially after rapidly overcoming the learning curve. Indeed, we observed that the operative time of R-ISR was heterogeneous among studies. Among the included studies, some surgeons may have less experiences of R-ISR as compared with L-ISR, which lead to a longer operative time in the R-ISR group. Another possible reason why the operative time was longer in the R-ISR group was that the robot can observe more lymph nodes in the low rectum with a more clear field of view as compared with laparoscope, thus increasing the time of lymph node dissection (21). Prolonged operative time can increase the risk of surgical site infection (SSI) (25) and may increase surgical team fatigue and room for more technical errors (26, 27). Regardless, it should be recognized that the duration of hospital stay was significantly lower in patients undergoing R-ISR than in those undergoing L-ISR, suggesting that the speed and quality of postoperative recovery should not be influenced by operative time in our meta-analysis.

Dissection of lymph nodes during radical surgery is related to the degree of radical resection and the survival and quality of life after surgery (28). Our meta-analysis showed that the mean number of lymph nodes harvested in patients undergoing R-ISR was a bit smaller than those undergoing L-ISR, but the difference was not significant between the two groups. There are some explanations for this unexpected phenomenon. First, the number of harvested lymph nodes is a parameter of the quality of the surgery and the minimum should be 12 lymph nodes for a correct pathological staging (29). It is pity that the number of lymph nodes harvested in the L-ISR of Baek's study was <12, which might cause the result inaccurate (14). Second, ISR surgery is more applicable for patients with T1 and T2 (30). Surgeons usually performed shorter resections with minimal lymph node dissection for this kind of tumors (31). Third, the scope of lymph node dissection may be smaller in the R-ISR group than in the L-ISR group. Moreover, there are a higher proportion of patients undergoing chemoradiotherapy before R-ISR, which might have affected the number of retrieved lymph nodes. In four of the included studies (14–17), more patients underwent chemoradiotherapy before R-ISR as compared with L-ISR.

The effect of the extent of anal sphincter resection on anal function is controversial among studies (32–35). Some studies suggested that anal function had no relationship with the extent of anal sphincter resection (32, 33), but others held the opposite view that the risk of fecal incontinence depended mainly on the height of the tumor and anastomotic site (34, 35). Notably, our included studies did not provide any relevant data regarding the extent of anal sphincter resection. Besides, J-type pouch coloanal anastomosis may be superior to direct anastomosis in protecting anal function (36). When anal function changes after surgery, anal lavage (37), biofeedback therapy (38), and sacral nerve stimulation therapy (39) can be used to promote the recovery of anal function.

The urinary function is mainly controlled by the sympathetic nerves from the superior hypogastric plexus and the parasympathetic nerves from the pelvic plexus and its branches (40). Surgical injury to the sympathetic nerve may lead to ejaculation dysfunction and injury to the parasympathetic nerve results in dysfunction of bladder detractor in male patients (41). Our meta-analysis showed that postoperative urinary complications occurred less frequently in the R-ISR group than in the L-ISR group. Because the mesorectum was anatomically in proximity to the pelvic nerves, it should be dissected as carefully as possible to reduce the damage of pelvic nerves (42). During the L-ISR surgery, it is often difficult to clearly identify subtle anatomical structures, probably increasing the risk of postoperative urinary dysfunction. By comparison, using a small and highly flexible robotic EndoWrist, the surgeons can more sufficiently expose the vascularless plane between the proper fascia of rectum and the anterior sacral fascia under the clear vision of the da Vinci robot. Considering a limited number of patients included in this study, more concrete evidence is needed to demonstrate the benefits of R-ISR on reproduction function over L-ISR.

This meta-analysis had several limitations. First, the data regarding the extent of anal sphincter resection, anastomosis methods, and neoadjuvant radiotherapy and chemotherapy were insufficiently reported, which prevented further subgroup analyses. Second, all included studies were non-randomized controlled studies with moderate quality. Third, the sample size is not adequate, and large-scale and multicenter randomized controlled studies are lacking to evaluate the long-term efficacy of R-ISR.

In conclusion, the potential benefits of R-ISR may be a safe and feasible choice for the treatment of low rectal tumors compared with L-ISR, including lower estimated intraoperative blood loss, postoperative urinary complications, and hospitalization days. However, high-quality large-scale randomized controlled trials are needed to compare R-ISR and L-ISR to guide the clinicians to choose the optimal approach for the treatment of low rectal tumors.

## Data Availability Statement

The original contributions presented in the study are included in the article/supplementary material, further inquiries can be directed to the corresponding author/s.

## Author Contributions

CZ involved in conceptualization. JZ, XQ, and CZ involved in methodology, data curation, and writing the original draft. XQ, FY, and CZ involved in validation. JZ, GG, FY, and CZ involved in formal analysis. GG, RC, and CZ involved in the investigation. XQ, FY, GG, and RC involved in writing the review and editing. CZ involved in supervision and project administration. All authors contributed to the article and approved the submitted version.

## Conflict of Interest

The authors declare that the research was conducted in the absence of any commercial or financial relationships that could be construed as a potential conflict of interest.

## Publisher's Note

All claims expressed in this article are solely those of the authors and do not necessarily represent those of their affiliated organizations, or those of the publisher, the editors and the reviewers. Any product that may be evaluated in this article, or claim that may be made by its manufacturer, is not guaranteed or endorsed by the publisher.

## Abbreviations

ISR: intersphincteric resection
R-ISR: robotic intersphincteric resection
L-ISR: laparoscopic intersphincteric resection
TME: total mesorectal excision
NOS: Newcastle-Ottawa Scale
RR: risk ratio
MD: mean difference
CI: confidence interval
BMI: body mass index
ASA: American Society of Anesthesiologists.
